# The Regentime stem cell procedure, successful treatment for a Charcot–Marie–Tooth disease case

**DOI:** 10.1002/ccr3.8358

**Published:** 2023-12-28

**Authors:** Nassim H. Abi Chahine, Vanessa J. Mansour, Lea I. Nemer, Cynthia F. Najjoum, Elsa A. El Asmar, Rita T. Boulos

**Affiliations:** ^1^ Regentime clinic Lebanon

**Keywords:** autologous stem cell therapy, bone marrow mononuclear progenitor stem cells, Charcot–Marie–Tooth disease, CMT, neurodegenerative disease, neurological disorder, peripheral neuropathy, PMP22, regenerative medicine, Regentime procedure

## Abstract

This report highlights the successful treatment of a Charcot–Marie–Tooth disease case using the Regentime stem cell procedure, suggesting its potential as a promising therapeutic approach for patients suffering from this challenging condition.

## 1 INTRODUCTION

Charcot‐Marie‐Tooth disease (CMT) is the most frequent form of inherited peripheral neuropathy, also referred to as hereditary motor and sensory neuropathy (HMSN). It is a phenotypically and genetically heterogeneous disorder affecting Schwann cells and/or axons, resulting in both motor and sensory manifestations.^1^ Medical pioneers including Virchow, Eulenburg, Friedreich, and Osler recorded cases of “peroneal muscular atrophy” prior to the seminal reports of the disease by Jean‐Marie Charcot and Pierre Marie from France and Henry Tooth from England. In 1886, Charcot, Marie, and Tooth independently described the clinical manifestations of HMSN which now carries their names. However, the term “peroneal muscular atrophy” was commonly used to describe CMT for over 100 years after the disease was first elucidated.^2^ CMT involves a spectrum of disorders arising from pathogenic variants in several genes encoding proteins that are expressed in myelin, gap junctions, and axonal structures within the peripheral nerves.^3^ There are several types of CMT, namely CMT types 1 through 7, in addition to CMTX which is an X‐linked type. This classification is further divided into subtypes that are defined by the mutated gene within each category and identified by letters (e.g., CMT1A, CMT1B, etc.).^4^

The relatively long size of a single neuron and its axon, often exceeding 1 m, contributes to its susceptibility to various genetic insults. The proper structural maintenance and function of peripheral nerves depends on the tightly regulated interactions between different organelles, synthesized proteins, and cargo molecules within the axons and Schwann cells.^4^, ^5^ Axons play an important role in the survival, proliferation, and differentiation of Schwann cells, which in turn are involved in regulating ion channels and supporting axonal survival and regeneration. This precisely regulated system within peripheral nerves is disrupted in CMT, leading to demyelination and/or axonopathy depending on the gene affected.^5^ Molecular and cellular mechanisms involved in the pathogenesis of different types of CMT include myelin assembly, cytoskeletal structure, endosomal sorting, vesicular transport, proteasome function, and mitochondrial regulation, among others.^4^, ^5^ The most common initial clinical features of CMT are slowly progressive distal symmetrical weakness, atrophy, and depressed deep tendon reflexes in the feet and legs, manifesting with foot drop and steppage gait. Upper extremities and proximal muscles of the lower extremities are affected later on in the course of the disease. Sensory signs and symptoms presenting as gradual loss of proprioception and vibration are less prominent and follow the same distal‐to‐proximal pattern over time.^2^, ^6^

The diagnostic evaluation involves clinical suspicion, electrophysiological studies, identification of inheritance patterns if present, genetic testing, and nerve biopsy in some cases. Electromyography (EMG) allows the categorization of CMT broadly into demyelinating and axonal types: homogeneously diffuse nerve conduction velocity slowing points to myelin dysfunction, whereas a normal or mildly reduced nerve conduction velocity with decreased muscle and sensory action potential amplitudes reveals an underlying axonopathy. Signs of denervation on needle EMG further indicate an axonal pathology. Some patients may present with both demyelinating and axonal electrophysiological features.^6^ Despite the ongoing research, there is currently no effective disease‐modifying treatment that changes the progressive course of CMT. Management relies predominantly on rehabilitation strategies to enhance functional abilities and mitigate the impact of the condition on daily life such as stretching, posture and balance exercises, aerobics, resistance training, and timely use of orthotic devices, in addition to surgical correction of foot deformities in some cases.^7^ We report the case of a 19‐year‐old male patient diagnosed with CMT at the age of 12. He had a slowly progressive symmetric sensory‐motor neuropathy resulting in sensory loss and gait difficulty requiring assistance. He underwent the Regentime procedure^8^ and restored normal gait and sensation 10 months following treatment. This presented case suggests the efficacy of autologous bone marrow‐derived partially in‐vitro differentiated mononuclear stem cell transplantation in promoting nervous system regeneration.

## 2 CASE PRESENTATION

A 19‐year‐old male patient was presented to our clinic for progressive bilateral lower limb muscle weakness and gradual sensory loss over 7 years. He reported an early history of frequent ankle sprains and difficulty running, which progressed to clumsy walking and ultimately led to a persistent need for a walker for ambulation. His family history was negative for hereditary neuropathy, and he underwent no genetic testing. He was diagnosed with CMT1 at the age of 16 based on clinical and electrodiagnostic cues. Upon physical examination, he had symmetrical lower limb atrophy, more evident distally. His motor power was registered to be 2/5 in his lower extremities distally and 4/5 proximally. He also had decreased pain, vibration, and proprioception, in particular disturbances in position sense. Moreover, he presented absent patellar and Achilles tendon reflexes. He had an overall neuropathy limitations scale (ONLS) of 4/12 as he had no functional disability involving his arms, but required bilateral support to walk 10 m. Otherwise, he had normal mental status, cranial nerves examination, upper limbs motor power, and cutaneous plantar reflexes. His EMG was done at the age of 16 years. Surface recording electrodes were placed over the abductor pollicis brevis and extensor digitorum brevis to estimate the motor conduction velocities in the median and peroneal nerves respectively. The median motor nerves conduction velocities were markedly reduced, reaching 20.2 m/s for the left median nerve and 26.5 m/s for the right median nerve, with decreased amplitudes. The extensor digitorum brevis muscles were found to be completely denervated; indicating an absence of responses for the peroneal nerves bilaterally. The left tibial nerve conduction study showed no response, while the right tibial nerve conduction study revealed a severely reduced conduction velocity of 17 m/s. The compound muscle action potential (CMAP) shapes were simple, and no conduction blocks were noted. The sensory nerve conduction study showed an absence of responses for the sural and median nerves. The findings of his EMG were in favor of diffuse demyelinating involvement of the peripheral nervous system, in line with the clinical suspicion of CMT1.

The patient signed an informed consent after providing him with an explanation about the Regentime stem cell procedure. He was then prepared for bone marrow collection by administering a total of three granulocyte colony‐stimulating factor doses. A bone marrow aspirate volume of 180 mL was collected (3 mL per Kg of body weight) and incubated at the stem cell laboratory. After 24 h of incubation, mononuclear stem cells underwent ultrafiltration, then re‐incubation with the Regentime differentiation agent, an organ‐specific proteinic ultrafiltrate. Progenitor cells were administered via two routes: intrathecally and intravenously. The patient was monitored for 2 days, during which he experienced neck stiffness of a moderate degree and reported mild headaches that were managed with paracetamol and non‐steroidal anti‐inflammatory drugs.

The patient was discharged on adenosine triphosphate, uridine, and cytidine monophosphate. Follow‐up examinations were held monthly after transplantation. The patient's motor power and sensation were gradually improving. At the 10th month, he was able to walk unassisted, had normal vibration and position senses, and restored normal patellar and Achilles tendon reflexes. His ONLS was noted to drop to 0/12. He reported feeling stronger and being able to perform daily activities with ease.

## 3 DISCUSSION

CMT is the most common inherited peripheral neuropathy, with a prevalence of 1 in 2500 individuals. Most of the patients exhibit symptoms during the first or second decade of their lives with slowly progressive length‐dependent weakness.^9^ CMT1 is a demyelinating form with an autosomal dominant pattern of inheritance showing gross nerve hypertrophy on nerve biopsy. Meanwhile, CMT2 is a dominantly inherited axonal neuropathy with axonal atrophy on nerve biopsy. CMT3 or Dejerine‐Sottas disease is a dominantly inherited severe early onset peripheral neuropathy that often presents with hypotonia during infancy. CMT4 has a recessive pattern of inheritance, often described in consanguineous families, and presents as rapidly progressive neuropathy during early childhood with extraneural features. CMT5 is an autosomal dominant neuropathy presenting with spasticity, CMT6 refers to patients with motor and sensory neuropathy associated with optic atrophy, and CMT7 refers to patients with motor and sensory neuropathy associated with retinitis pigmentosa.^4^ The majority of CMT cases are linked to pathogenic variants in four genes: PMP22, MPZ, GJB1, and MFN2 which constitute more than 90% of the molecular diagnoses.^4^, ^10^, ^11^

Neurophysiological studies categorize patients with CMT into demyelinating CMT1, characterized by a median motor nerve conduction velocity of less than 38 m/s, and axonal CMT2, with a preserved or mildly slowed median motor nerve conduction velocity of more than 38 m/s. This separation based on conduction velocities is clinically useful for proper diagnosis. The median motor nerve conduction velocity was chosen to be the reference over that of the peroneal motor nerve since the abductor pollicis brevis is less frequently totally denervated than the extensor digitorum brevis in CMT. An intermediate form is increasingly recognized in some patients presenting with mixed features, reflecting the heterogeneity of this disease.^6^, ^12^ Our patient began experiencing his symptoms early during the second decade of his life, complaining of slowly progressive distal symmetrical weakness and wasting in his lower limbs, along with sensory symptoms. His EMG showed a diffusely homogeneous and symmetrical peripheral nervous system demyelination with conduction velocities of <38 m/s in his median motor nerves, supporting the clinical suspicion of CMT1.^13^ He also had normal upper limb motor power and sensation despite evidence of electrophysiological abnormality, which can be compatible with a demyelinating form of CMT.^12^ The clinical severity of demyelinating CMT, such as the case in this report, correlates with the decrease in the CMAP amplitudes suggesting a secondary axonal degeneration as the major cause of signs and symptoms in CMT1.^3^

CMT1A is the most common subtype of CMT, accounting for 60%–90% of CMT1 cases, and around 50% of CMT patients. It is most commonly associated with a de‐novo duplication of the peripheral myelin protein 22 (PMP22) gene on chromosome 17, resulting in the overproduction of PMP22 and consequent demyelination.^3^, ^4^, ^13^ The physiological initiation and maintenance of myelination depends on the interaction between Schwann cells and axons. Timely synthesis of myelin constituents such as PMP22, P0, myelin basic protein, and myelin‐associated glycoprotein, in addition to their coordinated assembly and trafficking, play a major role in the regulation and maintenance of myelin structure. The trafficking of the PMP22 tetraspan glycoprotein from the endoplasmic reticulum and its insertion into the membrane is usually activated by axonal contact with the carboxy‐terminal of the protein.^14^ It is suggested that increased gene dosage of PMP22 coupled with rate‐limiting interactions with chaperones can lead to PMP22 misfolding above a critical threshold level resulting in abundant PMP22 degradation. In addition, misfolded PMP22 is thought to act as a chaperone‐like protein affecting intracellular physiological functions and normal regulation of myelination.^15^ Another suggested pathophysiological process of CMT1A is the increased apoptosis of Schwann cells including p53‐dependent apoptosis; however, the mechanisms responsible for this cell death are still unclear.^16^

There is currently no established treatment that can modify the progressive course of CMT.^7^ The application of the Regentime procedure for CMT is in line with autologous bone marrow‐derived mononuclear stem cells' neuroprotective, regenerative, and anti‐inflammatory properties. They secrete neurotrophic factors such as brain‐derived growth factor, glial‐derived growth factor, nerve growth factor, neurotrophin factor, ciliary neurotrophic factor, and basic fibroblast growth factor, which enable neuroprotection, prevent nerve degeneration and apoptosis, and promote neurogenesis including remyelination and axonal growth.^17^ Further, in‐vitro studies on the anti‐inflammatory mononuclear stem cells properties show that they can increase the production of growth differentiation factor‐15 and amphiregulin when cultured with PMP22‐overexpressing Schwann cells leading to the decrease in myelinating cells death and paving the way for the exploration of stem cell therapy for CMT, namely CMT1.^18^

The administration of drugs containing nucleotides such as adenosine triphosphate, uridine monophosphate, and cytidine monophosphate is shown to favor myelinated fibers regeneration and promote functional recovery in the setting of peripheral nervous system damage. The mechanisms involve the stimulation of neural cell protein synthesis, nerve cell membrane synthesis, myelin sheath synthesis, and neurite sprouting.^19^, ^20^ The importance of the above‐mentioned nucleotide supplementation, therefore, resides in its role in maximizing the efficacy of stem cell transplantation, thereby optimizing the overall outcome.^20^

To the best of our knowledge, this is the first CMT case treated with autologous bone marrow‐derived mononuclear stem cells globally. Our results underscore the potential of stem cell transplantation as a viable alternative approach in the treatment of CMT. However, large clinical trials are needed to confirm the efficacy of this therapy. Ultimately, continued research on the therapeutic effects of autologous stem cell transplantation in CMT can provide valuable insights into the underlying pathophysiological mechanisms, leading to more effective treatment options for patients.

## 4 CONCLUSION

CMT is a hereditary peripheral neuropathy that lacks a definitive therapeutic approach. We report the case of a CMT1 patient who recovered completely 10 months after autologous bone marrow‐derived mononuclear partially differentiated progenitor stem cell transplantation (the Regentime procedure). Our findings suggest that this stem cell transplantation technique could be a novel treatment modality for CMT. However, large clinical trials are needed to confirm its efficacy.

## AUTHOR CONTRIBUTIONS

**Nassim H. Abi Chahine:** Conceptualization; data curation; supervision; visualization; writing – review and editing. **Vanessa J. Mansour:** Writing – original draft. **Lea I. Nemer:** Writing – original draft. **Cynthia F. Najjoum:** Writing – original draft. **Elsa A. El Asmar:** Writing – original draft. **Rita T. Boulos:** Conceptualization; supervision; validation; writing – original draft; writing – review and editing.

## CONFLICT OF INTEREST STATEMENT

The authors declare that there is no conflict of interest regarding the publication of this work. They have no financial or personal relationships with any individuals or organizations that could potentially bias their findings or influence the content of this manuscript.

## CONSENT

Written informed consent was obtained from the patient to publish this report in accordance with the journal's patient consent policy.

## Data Availability

The data that supports the findings of this study are available upon request, ensuring the anonymity and confidentiality of the study participant. Any potentially identifying information will be appropriately redacted or anonymized to protect the privacy of our patient.
